# Case Report: SARS-CoV-2 Gamma Isolation From Placenta of a Miscarriage in Midwest, Brazil

**DOI:** 10.3389/fmed.2022.839389

**Published:** 2022-03-04

**Authors:** Zoraida Fernandez, Gislene G. C. Lichs, Claudia S. Zubieta, Ana B. Machado, Mia A. Ferreira, Natalia Valente, Thayssa Keren, Ighor Arantes, Valeria Nacife, Elisa Cavalcante Pereira, Luciana Appolinario, Thays E. J. Lacerda, Marilda M. Siqueira, Ana O. P. Esposito, Luiz H. F. Demarchi, Marina C. S. U. Zardin, Crhistinne C. M. Goncalves, Livia M. A. Maziero, Luciana A. F. Miziara, Felipe G. Naveca, Alex Pauvolid-Corrêa, Paola C. Resende, Alexsandra R. M. Favacho

**Affiliations:** ^1^Fundação Oswaldo Cruz (Fiocruz), Campo Grande , Brazil; ^2^Laboratório Central de Saúde Pública, Campo Grande , Brazil; ^3^Fundação Universidade Federal de Mato Grosso do Sul, UFMS, Campo Grande , Brazil; ^4^Rio de Janeiro State, Laboratório de Vírus Respiratórios e Sarampo da Fiocruz, Rio de Janeiro, Brazil; ^5^Secretaria de Estado de Saúde (SES), Campo Grande , Brazil; ^6^Centro de Informações Estratégicas de Vigilância em Saúde-CIEVS/SES, Campo Grande , Brazil; ^7^Centro de Informações Estratégicas de Vigilância em Saúde-CIEVS/Secretaria Municipal de Saúde de Campo Grande, Campo Grande , Brazil; ^8^Laboratório Ecologia de Doenças Transmissíveis na Amazônia, Instituto Leônidas e Maria Deane (Fiocruz Amazônia), Manaus, Brazil; ^9^Department of Veterinary Integrative Biosciences, Texas A&M University, College Station, TX, United States

**Keywords:** SARS-CoV-2, gamma variant, viral isolation, Brazil, placenta, fetal death

## Abstract

The present study investigated a SARS-CoV-2 infection in placenta and fetal samples from an early pregnancy miscarriage in Midwest Brazil. The Gamma variant was isolated and fully sequenced from the placenta sample, but not from fetal samples. Our findings highlight potential adverse perinatal outcomes caused by SARS-CoV-2 Gamma infection during pregnancy.

## Introduction

The emergence of the Severe Acute Respiratory Syndrome Coronavirus 2 (SARS-CoV-2) in December of 2019 in Wuhan, China, and its rapid worldwide spread resulted in a pandemic of coronavirus disease (COVID-19) with severe consequences for global public health (1). COVID-19 may vary from mild to severe and life-threatening conditions, especially in the elderly or patients with comorbidities (2).

Most COVID-19 patients have mild clinical signs and symptoms such as fever, cough, dyspnea, lymphopenia, and systemic effects (3). Pregnant women have an increased risk of developing pneumonia due to inherent physiology and immune response variations, which may lead to adverse obstetric and neonatal outcomes (4). COVID-19 during pregnancy may cause hypertensive disorders with placental inflammation distinct from typical preeclampsia (5). The placenta is usually an effective barrier that prevents fetuses' infection from spreading viruses. However, there is a theoretical risk of vertical transmission in SARS-CoV-2 since the ACE2 receptor is widely expressed in the organ, as reported for SARS (6). The use of antibody tests has provided new evidence that vertical transmission of SARS-CoV-2 can occur, and some studies have demonstrated an increase in perinatal death, mainly related to prematurity. Placenta samples taken from COVID-19 pregnant patients at mid-trimester and swabs and biopsies after spontaneous fetal loss at 19 weeks gestation have also tested positive for SARS-CoV-2 by molecular and immunohistochemical methods (5, 7). Fetal complications include miscarriage (2%), intrauterine growth restriction (10%), and premature birth (39%), and stillbirth, which is likely due to damage to the placenta (3).

Reports of SARS-CoV-2 infection during pregnancy remain limited, and the consequences of fetal infections caused by Variants of Concern (VOC) have been even less assessed. The present study describes virus isolation and whole-genome sequencing of SARS-CoV-2 Gamma variant from a placenta sample of an early pregnancy miscarriage.

## Methods

### Case Presentation

On February 22, 2021, a 31-week pregnant woman tested positive for SARS-CoV-2 by real-time RT-PCR performed in a private laboratory of Campo Grande, State of Mato Grosso do Sul (MS), Midwest Brazil. On February 23, she felt unwell and sought medical care and gestational follow-up. During clinical evaluation, the patient presented fever around 38°C, benign vital signs, headache, mild cough, eupneic, blood pressure of 110/70, heart rate of 78, and resting pulse oximetry of 99%. The fetal heart rate was 132 beats per minute, active and reactive. On February 25, a decreased fetal movement was reported during medical ultrasound examination, followed by fetal death. At the time of the miscarriage, the pregnant woman had not yet been vaccinated against SARS-CoV-2.

### SARS-CoV-2 Detection

Fetal nasopharyngeal swabs and placenta samples were sent to the Central Public Health Laboratory of MS (LACEN-MS) for further investigation. The RNA, of both samples, was extracted using Loccus and Extracta Fast Kit (MVXA-P096) and tested by a multiplex real time RT-PCR using the AllplexTM 2019-nCoV Assay (Seegene) and further confirmed by the multiplex EDx kit (Biomanguinhos/Fiocruz) (8). Subsequently, positive samples were submitted to a screening by real-time RT-PCR for detection targeting a deletion in the ORF1b gene (NSP6: S106del, G107del, F108del) found in the VOCs P.1 (Gamma); B.1.1.7 (Alpha) and B.1.351 (Beta) (9). For confirmation and complementary analysis, placenta, and fetuses' samples were sent to the Laboratory of Respiratory Viruses and Measles (LVRS) of Fiocruz, Brazil's National Reference Laboratory, and WHO Reference Laboratory for Coronavirus. At LVRS, positive samples were retested by real-time RT-PCR using the EDx kit (Biomanguinhos), and the CDC protocol (10). Positive samples were submitted to whole-genome sequencing and virus isolation in cell culture.

### Virus Isolation

The viral transport media (VTM) of the nasopharyngeal swab and a small fragment of the placenta were assayed in a biosafety laboratory level 3 (NB3), for viral cytopathic effect (CPE) on Vero E6 cell cultures as previously described (11). The placenta fragment was macerated, resuspended in *Dulbecco's Modified Eagle Medium* (DMEM), and clarified by centrifugation for inoculation. Samples were inoculated in Vero cells seeded in 25 cm^2^ flasks and maintained at 37°C with 5% CO_2_ for 1 h to optimize virus adsorption. Then, maintenance media supplemented by 2% FBS was added, and cell cultures were inspected daily under an inverted microscope for CPE in a total of two 4-day blind passages.

### Sample Sequencing

Sequencing was conducted using Illumina protocols previously established and used by the Fiocruz COVID-19 Genomic Surveillance Network to acquire high-quality genomes (P.C. Resende, unpub. data, https://doi.org/10.1101/2020.04.30.069039). The FASTQ reads obtained were imported into the CLC Genomics Workbench version 20.0.4 (QIAGEN), trimmed, and mapped against the reference sequence EPI_ISL_402124 (hCoV-19/Wuhan/WIV04/2019) available in EpiCoV database in the GISAID (https://www.gisaid.org/). The contig was refined using the InDels and Structural Variants module then, the Local Realignment module and the final consensus were obtained. The SARS-CoV-2 lineage characterization was performed by Pango Lineage (12). To characterize phylogenetically both sequences, a maximum likelihood tree was inferred with a dataset composed of various SARS-CoV-2 lineages circulating in Brazil (*n* = 189). The tree was constructed with IQ-TREE v. 2.1.3 (13), and statistical support of its topology estimated with the approximate likelihood-ratio test (aLRT) (14). The nucleotide and amino acid features were observed by the online tools NextClade (https://clades.nextstrain.org/) and CoVSurver (https://mendel.bii.a-star.edu.sg/METHODS/corona/beta/).

## Results

**Figure 1:**
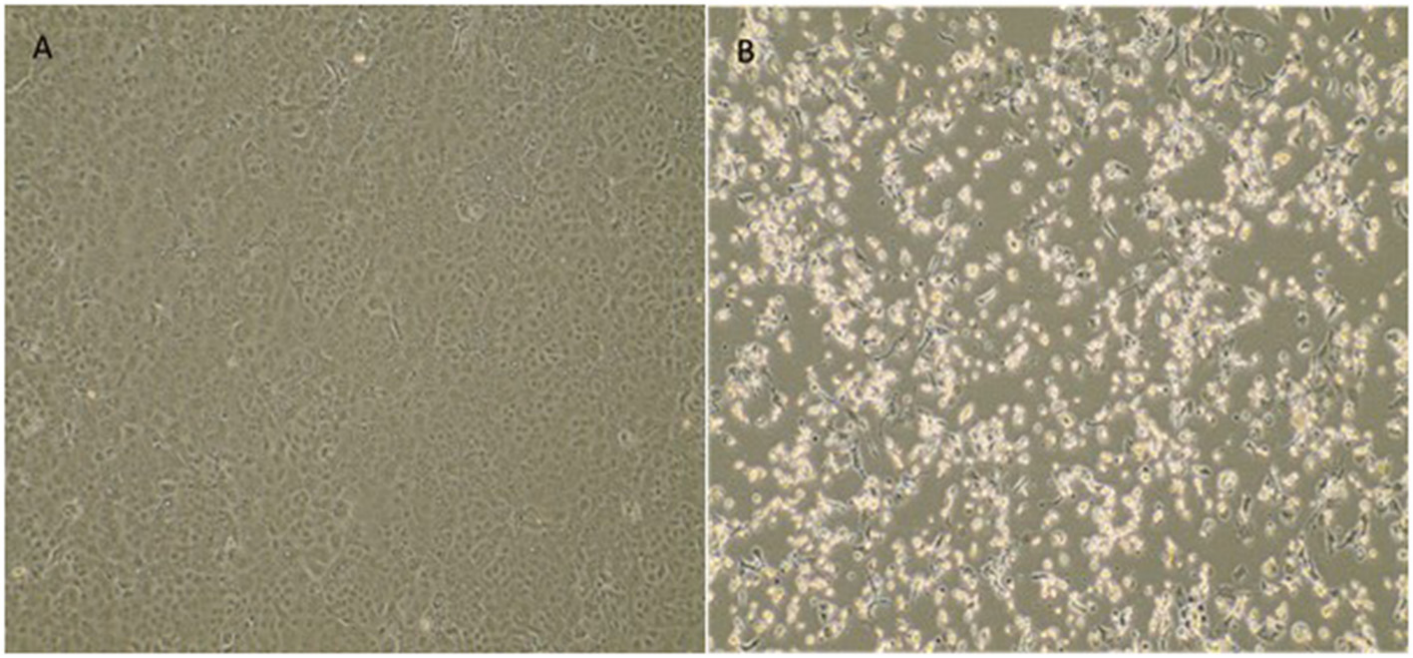
Cytopathic effect of SARS-CoV-2 variant of concern Gamma (P.1) from a placenta sample in Vero E6 cell culture. **(A)** Vero E6 cell cultures (negative control). **(B)** Cytopathic effects consisting of rounding and detachment of cells in VERO E6 cultures 3 days after the third passage. EVOS™ XL Core Imaging System—Thermo Fisher Scientific (10×).

**Figure 2:**
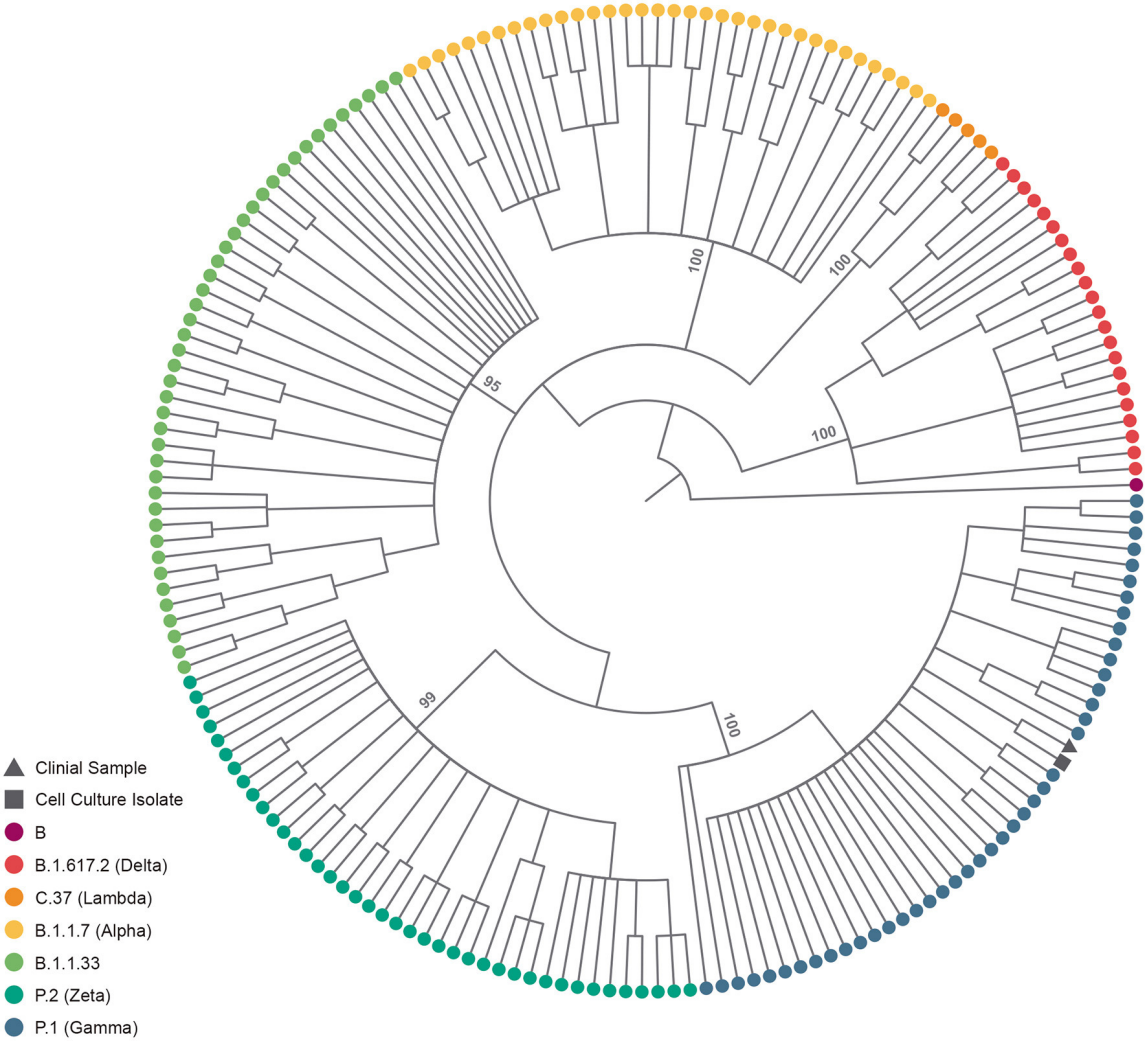
SARS-CoV-2 lineage assignment of placenta sample and its cell culture isolate. Cladogram-transformed maximum likelihood tree (*n* = 191) of SARS-CoV-2 complete genomes (*n* = 29,412). The tree encopasses Brazillian sequences of PangoLineage designated B.1.617.2 (*n* = 22) VOC Delta, C.37 (*n* = 5) VOI Labmda, B.1.1.7 (*n* = 35) VOC Aplha, B.1.1.33 (*n* = 43), P.2 (*n* = 40) VOI Zeta and P.1 (*n* = 45) VOC Gamma, identified by their tip colors. The clinical sample (EPI_ISL_2274091) and cell culture isolate (EPI_ISL_2863796) are identified by their tip shapes. Statistical support (approximate likelihood-ratio test, aLRT) of lineage branches is annotated in the tree.

The fetal nasopharyngeal swabs were negative for SARS-CoV-2 by real time RT-PCR, but the placenta sample was SARS-CoV-2 positive by real time RT-PCR (Table 1) and the virus was isolated. CPE caused by SARS-CoV-2 was confirmed by real-time RT-PCR of culture supernatant. In cases where no CPE was observed, real-time PCR was performed four days after inoculation to confirm the absence of virus replication (Figure 1). Whole-genome sequencing confirmed infection by SARS-CoV-2 Gamma lineage confirming the deletions at the ORF1a gene (9). The obtained nucleotide sequences of the placenta fragment (EPI_ISL_2274091) and the cell culture isolate (EPI_ISL_2863796) (Figure 1) were subsequently used alongside a panel of Brazilian sequences of SARS-CoV-2 lineages in the inference of a maximum likelihood tree (Figure 2). Both samples branched among highly supported (aLRT = 100) Gamma lineage branches, corroborating their Pango Lineage classification (12).

**Table 1:** Real time RT-PCR for SARS-CoV-2 in samples from an early pregnancy miscarriage in Midwest Brazil.

**Protocol**	**Allplex (Seegene)**			**CDC**		**EDx (Biomanguinhos)**	**Variants**
Targets	E	RdPD	N	N1	N2	E	
Nasopharyngeal swab (pregnant)	ND^*^	ND^*^	ND^*^	NT	NT	ND^*^	NT^**^
Placenta	18	14	14	NT	NT	22	17
Virus isolated in Vero cells (placenta)	NT	NT	NT	10,2	10,6	10.1	NT^**^
Nasopharyngeal swab (fetus)	ND^*^	ND^*^	ND^*^	ND^*^	ND^*^	ND^*^	NT^**^

* *ND, not detected;*

** *NT, not tested*.

## Discussion

An analysis of a subset of viral sequences from the consortium Fiocruz COVID-19 Genomics Surveillance Network of the Brazilian Ministry of Health (http://www.genomahcov.fiocruz.br/) and the Federal University of state of Mato Grosso do Sul revealed that between March and April 2021, 68 (83%) out of 82 SARS-CoV-2 sequences from MS were VOC Gamma. Additionally, an assessment of the molecular composition of both sequences by the NextClade algorithm revealed that as they bare all synapomorphic signatures of the VOC Gamma, two additional amino-acid substitutions are shared, both in ORF1a, P885L and T2121I.

Here, we report a case of COVID-19 during an early pregnancy miscarriage confirmed by virus isolation and the SARS-CoV-2 whole-genome sequencing of placenta samples. The placenta is an effective maternal-neonatal barrier against the virus even in the presence of a severe infection. However, the placental damage induced by the SARS-CoV-2 may have detrimental effects for the neonate independently of vertical transmission (15).

Despite the increasing number of published studies on COVID-19 infection during pregnancy, other studies are needed to better understand the effect of different variants of the virus on pregnant women and the complications they may develop. Data from clinical studies and systematic review (16–19) shown the effect of SARS-CoV-2 infection in terms of maternal and fetal outcomes. The data have shown that SARS-CoV-2 infection in pregnancy, when compared with non-pregnant women, is asso-ciated with a small increase in risk to the mother, and the most common clinical features are fever, cough, myalgia, and shortness of breath (16, 19). However, there are reports of cases of women who developed severe disease and needed to be admitted to ICU (16). The recommendations for pregnant women with COVID-19 are based on previous experiences with SARS-CoV and MERS-CoV infections. Some of principles of management include early isolation, oxygen therapy, avoidance of fluid overload, antibiotics for secondary bacterial infection risk, fetal and uterine contraction monitoring, and early mechanical ventilation for progressive respiratory failure. To prevent the infection by the virus it is advisable avoid crowed areas, postpone those medical consultations that are not essential and if it is possible consult the doctor virtually. The vaccination against SARS-CoV-2 is of great importance to reduce risks of serious illness, premature birth, and abortions (20).

## Data Availability Statement

The datasets presented in this study can be found in online repositories. The names of the repository/repositories and accession number(s) can be found in the article/supplementary material.

## Ethics Statement

This study was approved by the FIOCRUZ-IOC Ethics Committee (68118417.6.0000.5248 and CAAE 32333120.4.0000.5190) and the Ministry of Health of Brazil SISGEN (A1767C3). The patients/participants provided their written informed consent to participate in this study. Written informed consent was obtained from the individual(s) for the publication of any potentially identifiable images or data included in this article.

## Author Contributions

All authors listed have made a substantial, direct, and intellectual contribution to the work and approved it for publication.

## Conflict of Interest

CG was employed by Secretaria de Estado de Saúde (SES). The remaining authors declare that the research was conducted in the absence of any commercial or financial relationships that could be construed as a potential conflict of interest.

## Publisher's Note

All claims expressed in this article are solely those of the authors and do not necessarily represent those of their affiliated organizations, or those of the publisher, the editors and the reviewers. Any product that may be evaluated in this article, or claim that may be made by its manufacturer, is not guaranteed or endorsed by the publisher.
